# Regulatory Effects of CD14^+^CD16^++^Slan^+^ Monocytes on Classical Monocytes and T Lymphocytes in Response to Inflammatory and Phagocytic Stimuli

**DOI:** 10.1155/jimr/3322498

**Published:** 2026-04-11

**Authors:** Cristian A. Anacona, Karen Álvarez, Gloria Vásquez, Mauricio Rojas

**Affiliations:** ^1^ Cellular Immunology and Immunogenetics Group, Faculty of Medicine, University of Antioquia, Medellín, Colombia, udea.edu.co; ^2^ Rheumatology Section, San Vicente Fundación University Hospital, Medellín, Colombia; ^3^ Flow Cytometry Unit, University Research Center, University of Antioquia, Medellín, Colombia, udea.edu.co

**Keywords:** apoptotic bodies, cytokine modulation, monocyte subsets, Slan^+^ monocytes, T cell proliferation

## Abstract

**Background:**

CD14^+^CD16^++^6‐sulfo LacNAc (Slan)^+^ monocytes represent a specialized nonclassical subset with reported pro‐inflammatory and regulatory features. However, their capacity to modulate classical monocytes and T lymphocytes under apoptotic and inflammatory conditions remains incompletely defined.

**Methods:**

We employed a coculture reconstitution model using flow cytometry—sorted CD14^+^CD16^++^Slan^+^ monocytes and CD14^++^CD16^-^Slan^−^ classical monocytes at defined ratios. Cultures were stimulated with lipopolysaccharide (LPS) or K562‐derived apoptotic bodies (ABs). Monocyte surface marker expression was evaluated by flow cytometry, and cytokine concentrations were quantified using multiplex bead‐based assays. The impact of Slan^+^ monocytes on CD3^+^ T cell proliferation and cytokine production was assessed following monocyte–T cell coculture.

**Results:**

CD14^+^CD16^++^Slan^+^ monocytes exhibited enhanced uptake of ABs and distinct phenotypic modulation upon stimulation. Increasing proportions of Slan^+^ cells induced higher HLA‐DR and CCR5 expression on classical monocytes. LPS stimulation promoted TNF, IL‐12, IL‐6, and CCL2 production, whereas AB exposure favored IL‐10 and IL‐17 accumulation. In coculture assays, Slan^+^ monocytes reduced CD4^+^ and CD8^+^ T cell proliferation and altered T cell cytokine profiles in a stimulus‐dependent manner.

**Conclusion:**

CD14^+^CD16^++^Slan^+^ monocytes exert a dose‐ and context‐dependent modulatory effect on classical monocytes and T lymphocytes in vitro, displaying both inflammatory and regulatory properties depending on the stimulus. These findings highlight their functional plasticity within the human monocyte compartment.

**Summary**

• 6‐sulfo LacNAc (Slan)^+^ monocytes actively internalize apoptotic bodies (ABs) and show high phagocytic activity.
• Coculture with Slan^+^ monocytes increases HLA‐DR and CCR5 expression in classical monocytes.
• Cytokine secretion profiles vary depending on Slan^+^ cell proportion and stimulus (lipopolysaccharide [LPS] vs. ABs).
• Slan^+^ monocytes suppress CD4^+^ and CD8^+^ T cell proliferation and modulate cytokine output.
• This subset shows dual plasticity, promoting both pro‐inflammatory and regulatory responses.

## 1. Introduction

Human monocytes, derived from bone marrow precursors, are key components of the innate immune system. They are primarily involved in phagocytosis, cytokine production, and antigen presentation. Based on the differential expression of CD14, a pattern recognition receptor, and CD16, an FcγIII receptor, monocyte subsets were first described by Passlick et al. [1], revealing distinct morphological and functional profiles between the classical (CD14^++^CD16^−^) and nonclassical (CD14^−^CD16^+^) populations [2, 3].

An intermediate monocyte population (CD14^+^CD16^+^HLA‐DR^+^CD86^+^CD11c^+^) was subsequently identified and transcriptionally characterized as a distinct subset enriched in genes associated with antigen processing and inflammatory regulation, including LYZ, S100A8, CD74, HLA‐DRA, and IFI30 [4]. More recently, comprehensive phenotypic and transcriptomic analyses have demonstrated that the 6‐sulfo LacNAc (Slan) epitope, detected by the M‐DC8 antibody, identifies the prototypical nonclassical CD16^+^ monocytes in human blood rather than a dendritic cell lineage [5]. These Slan^+^ cells display a stable nonclassical monocyte transcriptional signature, high CD16 expression, reduced CD14 levels, and a distinct pro‐inflammatory competence characterized by robust TNF‐α and IL‐12 production in response to TLR stimulation. This reclassification has positioned CD14^+^CD16^++^Slan^+^ monocytes as a functionally specialized subset within the nonclassical compartment, with defined migratory, antigen‐presenting, and cytokine‐producing capacities [5].

Although initially considered Slan^+^ dendritic cells due to their potent cytokine secretion and use of IgMκ‐conjugated paramagnetic beads for their isolation, further analyses confirmed that these M‐DC8^+^/Slan^+^ cells exhibit high CD16 and low CD33 expression and cluster transcriptionally and functionally with monocytes, not with CD1c^+^ dendritic cells [6, 7]. Consequently, these cells are now recognized as a distinct subset of nonclassical monocytes, referred to hereafter as CD14^+^CD16^++^Slan^+^ monocytes.

This subset plays an active role in diverse pathological conditions. For instance, increased frequencies of CD16^+^Slan^+^ monocytes have been reported in the kidneys of systemic lupus erythematosus (SLE) patients with lupus nephritis, suggesting their involvement in immune complex‐mediated glomerular injury [8]. In oncology, their blood levels rise in patients with colorectal carcinoma and diffuse large B‐cell lymphoma, while their presence in tumor‐draining lymph nodes and absence in primary or metastatic tumor sites suggest a context‐dependent function [9, 10]. Additionally, Slan^+^ cells mediate CD20‐dependent B‐cell lymphoma elimination via “Antibody‐Dependent Cellular Cytotoxicity (ADCC) and Antibody‐Dependent Cellular Phagocytosis (ADCP)” [10].

Previous studies have indicated that CD14^+^CD16^++^ monocytes can regulate the function of classical monocytes, modulate their response to apoptotic bodies (ABs), and affect mitogen‐induced T cell proliferation. However, their function appears altered in SLE, where these monocytes exhibit a dysfunctional phenotype potentially linked to tolerance loss [11]. Notably, the functional distinction between CD16^+^Slan^+^ and CD16^+^Slan^−^ subsets remains unclear.

In this study, we aimed to dissect the functional interplay between CD14^+^CD16^++^Slan^+^ monocytes and their classical CD14^++^CD16^−^ counterparts under immunoregulatory (ABs) and pro‐inflammatory (lipopolysaccharide [LPS]) stimuli. We established a coculture model using highly purified Slan^+^ and Slan^−^ monocytes at defined ratios to assess their phenotypic and functional responses, including surface marker expression, cytokine secretion, and modulation of autologous T lymphocyte proliferation.

## 2. Materials and Methods

### 2.1. Study Population

This experimental study included healthy adult volunteers who provided written informed consent. To be considered in this study, we excluded individuals with a history of cancer, diabetes mellitus, autoimmune diseases (including SLE and rheumatoid arthritis), recent infections, or use of immunosuppressants, antibiotics, or hormones. Each experiment used venous blood samples (60 mL) from five independent donors. The Ethics Committee of the School of Medicine, Universidad de Antioquia, approved the study protocol.

### 2.2. Ethical Approval

This study was conducted in accordance with the Declaration of Helsinki and approved by the Ethics Committee of the School of Medicine, Universidad de Antioquia (Approval Code: 2019‐IRB‐063). All participants provided written informed consent prior to sample collection.

### 2.3. Whole Blood Immunophenotyping by Flow Cytometry

Peripheral venous blood from healthy donors was collected in EDTA‐containing tubes and processed within 2 h of collection. For surface immunophenotyping, 30 µL of whole blood was incubated for 20 min at room temperature in the dark with pretitrated fluorochrome‐conjugated monoclonal antibodies against CD14, CD16, Slan (M‐DC8), CCR5, CD68, CD86, TLR4, HLA‐DR, and CD69.

Following antibody staining, red blood cells were lysed using Optylyse C (Beckman Coulter) according to the manufacturer’s protocol. After lysis, isotonicity was restored by adding deionized distilled water. Samples were acquired without washing to minimize cell loss.

Data were acquired on an LSR Fortessa II flow cytometer (BD Biosciences) equipped with multiple lasers and standard optical configurations. Instrument performance and photomultiplier tube (PMT) voltages were standardized using daily cytometer setup and tracking (CST) procedures. Compensation was calculated using BD CompBeads individually stained with each fluorochrome‐conjugated antibody. Fluorescence‐minus‐one (FMO) controls were used when necessary to define gating boundaries.

Monocytes were identified based on forward scatter (FSC‐A) and side scatter (SSC‐A) characteristics, followed by doublet exclusion using FSC‐A vs. FSC‐H gating. CD14 and CD16 expressions were used to define classical (CD14^++^CD16^-^), CD16^+^ (CD14^+^CD16^+^/^++^), and Slan^+^ (CD14^+^CD16^++^Slan^+^) subsets. Median fluorescence intensity (MFI) values were calculated for each marker within the gated populations (Figure S1 defines the gating strategy to define Slan+ monocytes).

Data were analyzed using FlowJo software (Version 10.1.0), and results are expressed as MFI with corresponding statistical analysis as indicated.

### 2.4. Isolation of Peripheral Blood Mononuclear Cells (PBMCs)

PBMCs were isolated from EDTA‐anticoagulated blood via density gradient centrifugation using Histopaque (*ρ* = 1.077 g/L). Cells were washed twice in PBS and resuspended in RPMI‐1640 medium. Viability was assessed by trypan blue exclusion, with viable cells exceeding 90%.

### 2.5. Generation of ABs

ABs were generated from the K562 cell line (ATCC CCL‐243), cultured at 1 × 10^6^ cells/mL in RPMI‐1640 supplemented with 10% heat‐inactivated fetal bovine serum (FBS), 100 μg/mL streptomycin, 100 U/mL penicillin, and 1% L‐glutamine. After 48 h of expansion at 37°C in 5% CO_2_, apoptosis was induced by culturing cells in PBS containing 1 μg/mL colchicine for an additional 48 h, following an adapted protocol [12].

### 2.6. Rationale for Using K562‐Derived ABs and Immunogenicity Controls

K562 cells were selected as a standardized and reproducible source of apoptotic material across donors. Importantly, K562 cells show negligible basal surface expression of HLA class I and class II molecules, which reduces the likelihood that apoptotic material provides alloantigens capable of driving allogeneic activation in our autologous coculture system. This choice therefore minimizes confounding allo‐recognition while allowing controlled delivery of apoptotic cargo to monocytes. In our setting, K562 cells were not exposed to interferons or other differentiation stimuli known to upregulate HLA expression, and ABs were generated from sorted DiOC6^low^ propidium iodide (PI)^−^ cells to enrich for apoptotic events and exclude necrotic contaminants [13].

In addition, to reduce the contribution of pro‐inflammatory DAMPs associated with nonapoptotic death, the AB preparation was validated for phosphatidylserine exposure, membrane integrity, and lack of HMGB1 staining after thawing (data not shown).

Cells were then stained with DiOC6 (700 nM) and PI (1 μg/mL) to detect mitochondrial and membrane damage. DiOC6^low^ PI^−^ cells were sorted by flow cytometry (FACSAria III, BD Biosciences), yielding >99% purity. ABs were frozen in RPMI‐1640 with 10% DMSO and 50% FBS, stored in liquid nitrogen, and later validated for phosphatidylserine exposure, membrane integrity, and absence of HMGB1 expression.

### 2.7. Monocyte Subset Isolation by Flow Cytometry

Ten million PBMCs were stained with anti‐CD14‐PE, anti‐CD16‐FITC, anti‐Slan‐VioBlue (M‐DC8 clone DD1), and anti‐CD45‐PE‐Cy7. Monocytes were gated as CD45^high^ cells with low SSC and appropriate CD14/CD16 profiles. CD14^++^CD16^−^ Slan^−^ (classical) and CD14^+^CD16^++^Slan^+^ (nonclassical) monocytes were sorted into separate populations using FACSAria III under sterile conditions. Purity exceeded 93% for both subsets, with a recovery efficiency of more than 89%.

All flow cytometry experiments were conducted in accordance with current best practices. Compensation controls were performed using BD CompBeads (BD Biosciences) for each fluorochrome‐conjugated antibody. Single‐stain controls verified the accuracy of the compensation matrix for cells, ensuring that the signals were separated correctly. Cytometer performance was validated daily using BD CST beads for the FACSAria III instrument and quality control (QC) beads for the CytoFLEX cytometer (Beckman Coulter). Instrument settings were maintained within recommended target values. All acquisition and analysis procedures were conducted in accordance with MIFlowCyt guidelines to ensure standardization, reproducibility, and data integrity.

Because Slan identifies a minor subset within CD16^+^ monocytes, high‐purity isolation of Slan^+^ cells require direct labeling and FACS sorting; negative‐selection approaches do not provide a practical route to separate Slan^+^ from Slan^-^ fractions. Although CD16^+^ monocytes can be enriched by additional steps, implementing a two‐step enrichment‐plus‐sorting workflow increases ex vivo manipulation time and mechanical stress, decreases yield and viability, and may compromise cell quality. Therefore, both Slan^+^ and Slan^−^ monocytes were isolated by the same antibody‐based sorting strategy under identical conditions to minimize differential handling effects.

### 2.8. Coculture and Reconstitution Experiments

Sorted Slan^+^ and Slan^−^ monocytes were seeded at defined ratios (0:100, 10:90, and 40:60) in 96‐well flat‐bottom plates (1 × 10^5^ total cells/well). Cells were initially adhered to RPMI‐1640 with 0.5% FBS for 4 h, then replaced with RPMI‐1640 containing 10% FBS. Slan^−^ cells were prelabeled with CFSE to track the responses of subpopulations separately [14]. Slan^+^ monocytes constitute a minor fraction of circulating monocytes in vivo (~5%–10% of total monocytes). Accordingly, a 9:1 (Slan^−^:Slan^+^) ratio was selected to approximate physiological predominance under steady‐state conditions. The 6:4 ratio modeled a relative enrichment scenario to evaluate whether shifts in subset balance modify functional outcomes. These ratios were chosen to explore changes in relative abundance rather than to reproduce a single fixed in vivo proportion.

### 2.9. Stimulation With LPS and ABs

Cultures were stimulated with either LPS (1 μg/mL) or ABs (five ABs per monocyte) [11, 12] and incubated for 48 h at 37°C in 5% CO_2_. Surface marker expression (HLA‐DR, CD80, CD86, CD69, CCR5, CD68, and TLR4) was assessed by flow cytometry in both monocyte subpopulations (Slan^+^ and Slan^−^ monocytes). Cell viability was determined prior to analysis using Zombie Violet live/dead discrimination dye (excitation 405 nm, emission 525 nm with a 40‐nm bandpass filter). The final number of cells were always 1 × 10^5^ total cells/well.

### 2.10. Cytokine Quantification

At 48 h, 50 μL of supernatant from each culture was stored at −70°C and analyzed for TNF‐α, IL‐1β, IL‐6, IL‐10, IL‐12, IL‐23, and CCL2 concentrations using the Luminex 200 system. Cytokine levels (pg/mL) were calculated using xPONENT software and standard curves per the manufacturer’s instructions [11].

### 2.11. AB Internalization Assay

To assess AB uptake, 1 × 10^5^ Slan^+^ or Slan^−^ monocytes were seeded on poly‐L‐lysine‐coated 12 mm glass coverslips. After 2 h of adherence, cells were incubated with CFSE‐labeled ABs (3 ABs/monocyte) and 2 μM LysoTracker Red for 2 h, followed by Hoechst nuclear staining. Samples were fixed in FluorSave and imaged using EVOS M5000 fluorescence microscopy. Colocalization of LysoTracker, CFSE, and Hoechst signals was analyzed with ImageJ software, calculating Pearson and Manders coefficients.

Pearson correlation coefficients shown in Figure 1B were calculated from fluorescence images obtained from five independent donors. For each donor, multiple nonoverlapping microscopic fields were acquired using identical acquisition settings (laser intensity, gain, and exposure time) to avoid signal saturation and ensure comparability across samples. Fields were selected according to predefined systematic sampling criteria to minimize selection bias.

Figure 1Internalization of K562‐derived apoptotic bodies (ABs) by Slan^+^ and Slan^−^ monocytes. (A) Representative fluorescence microscopy images of FACS‐purified Slan^+^ and Slan^−^ monocytes cultured on poly‐L‐lysine‐coated coverslips and incubated for 2 h with CFSE‐labeled apoptotic bodies (three ABs per monocyte). Lysosomes were stained with LysoTracker Red (2 μM), nuclei with Hoechst 33342, and images acquired using an EVOS M5000 inverted fluorescence microscope (40× objective). Colocalization was analyzed using ImageJ software. Pearson’s correlation coefficient, Manders’ overlap coefficients (M1 and M2), and Li’s intensity correlation quotient (ICQ) were calculated for each cell. (B) Quantitative analysis of Pearson’s correlation coefficient from independent donors (*n* = 5 biological replicates; ≥20 cells analyzed per donor). Data are shown as mean ± SEM. Statistical significance was assessed using a two‐tailed Student’s *t*‐test after confirming normal distribution. *p* = 0.0011. Scale bars: 10 μm.  ^∗∗^ *p* < 0.005 (*p* exactly equal to 0.0011).

**Figure:**
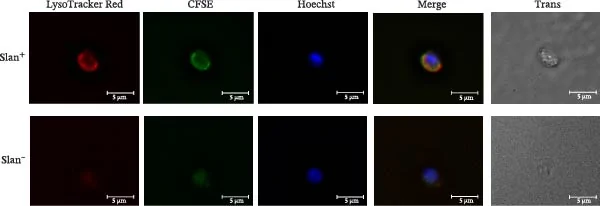
(A)

**Figure:**
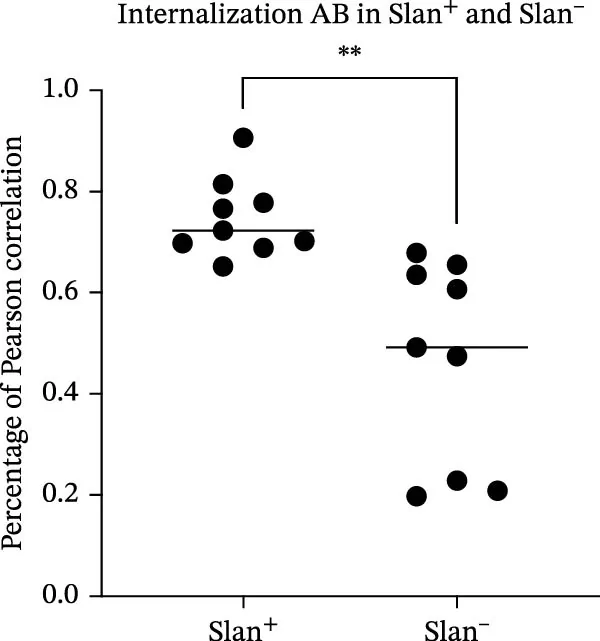
(B)

Colocalization analysis was performed using the Coloc2 plugin in ImageJ (NIH). Background subtraction was applied uniformly, and thresholding was performed using an automated Costes method to ensure objective quantification. Pearson coefficients were calculated for each field, and values were averaged per donor prior to statistical analysis to avoid pseudoreplication. The nine measurements displayed correspond to independent image fields distributed across the five donors.

### 2.12. T Cell Cocultures and Proliferation Assays

Autologous CD3^+^ T cells were isolated magnetically (>95% purity), labeled with CFSE, and cocultured with monocyte mixtures at a 3:1 lymphocyte: monocyte ratio. As a proliferation control, PHA‐L (5 μg/mL) was added to selected wells. After 120 h, cells were stained with Zombie Violet, anti‐CD4, and anti‐CD8 antibodies. CFSE dilution was analyzed to assess proliferation. Supernatants were collected for cytokine analysis (IL‐2, IFN‐γ, IL‐13, IL‐10, and IL‐17) using Luminex.

## 3. Statistical Analysis

Statistical analyses were performed using GraphPad Prism version 8.0 (GraphPad Software, San Diego, CA, USA). Normality and homoscedasticity were evaluated using the Shapiro–Wilk and Levene’s tests, respectively. As most datasets did not follow a normal distribution, comparisons among groups were conducted using the nonparametric Kruskal–Wallis test followed by Dunn’s multiple comparison post hoc test with Bonferroni adjustment when appropriate. Trend analyses and slope comparisons were additionally performed to assess dose‐dependent effects of increasing proportions of Slan^+^ monocytes. Data are presented as mean ± 95%CI. A *p* value < 0.05 was considered statistically significant.

## 4. Results

### 4.1. Isolation and Characterization of Monocyte Subsets

Using a previously validated gating strategy [7], CD14^++^CD16^−^Slan^−^ (classical) and CD14^+^CD16^++^Slan^+^ (nonclassical) monocytes were successfully isolated from healthy donors via flow cytometry. The resulting populations showed purities ranging from 93% to 98% and recovery efficiencies above 89%. All Slan^+^ and Slan^−^ cells expressed HLA‐DR, consistent with their antigen‐presenting function.

### 4.2. Intrinsic Basal Phenotypic Differences Between Slan^+^ and Slan^−^ Circulating Monocytes

To determine whether Slan^+^ monocytes (Figure S1 defines the gating strategy to define Slan+ monocytes) display intrinsic phenotypic differences under steady‐state conditions, the basal expression of activation‐ and differentiation‐associated markers was analyzed directly ex vivo in whole blood from healthy donors. Representative histograms (Figure S2A) illustrate distinct expression patterns across classical (CD14^++^CD16^-^Slan^−^), CD16^+^Slan^−^, and CD14^+^CD16^++^Slan^+^ monocytes. Quantitative analysis of MFI confirmed significant subset‐specific differences (Figure S2B).

HLA‐DR expression showed marked stratification among subsets. CD16^+^Slan^−^ monocytes expressed significantly higher HLA‐DR levels compared to both Slan^+^ monocytes (*p* < 0.01) and classical CD14^++^CD16^-^ cells (*p* < 0.01), indicating a more pronounced basal antigen‐presenting phenotype within the CD16^+^Slan^-^ compartment. TLR4 expression was modest but significantly increased in classical CD14^++^CD16^-^ monocytes compared to Slan^+^ cells (p < 0.05), suggesting differential basal sensitivity to LPS among subsets.

CCR5 and CD86 displayed subset‐dependent trends without reaching statistical significance, although CD16‐expressing populations tended to exhibit higher expression levels compared to classical monocytes. Notably, CD68 expression differed significantly between Slan^+^ and classical monocytes (*p* < 0.01), with CD16‐expressing subsets showing increased levels relative to CD14^++^CD16^−^ cells, consistent with a more activation‐associated basal phenotype. CD69 expression remained comparable across subsets and did not show significant differences, supporting the absence of overt systemic activation in healthy donors.

Collectively, these findings demonstrate that Slan^+^ monocytes exhibit a defined basal molecular profile distinct from both classical and CD16^+^Slan^−^ monocytes. Importantly, these differences are detectable in vivo under steady‐state conditions, providing mechanistic context for the functional divergence observed in subsequent stimulation and coculture experiments.

### 4.3. Uptake of ABs by Slan^+^ Monocytes

Microscopy analysis revealed significantly greater uptake and lysosomal processing of CFSE‐labeled ABs in Slan^+^ monocytes compared to Slan^−^ monocytes. Slan^+^ cells displayed higher colocalization of LysoTracker Red and CFSE signals (mean Pearson coefficient = 0.85) than Slan^−^ cells (mean Pearson coefficient = 0.48) (Figure 1). These data indicate that Slan^+^ monocytes possess enhanced phagocytic and metabolic activity toward ABs.

### 4.4. Differential Expression of Differentiation Markers

#### 4.4.1. Phenotypic Stability of Slan^+^ and Slan^−^ Monocytes After 48 h in Unstimulated Culture Conditions

As shown in Figure S3, to determine whether intrinsic phenotypic differences between Slan^+^ and Slan^-^ monocytes persist after short‐term in vitro culture, both subsets were maintained for 48 h under unstimulated (NS) conditions and analyzed for surface marker expression.

As shown in Figure S3, subset‐specific differences observed ex vivo were not completely abolished after 48 h. Slan^+^ and Slan^−^ monocytes maintained significantly distinct expression levels of several activation‐ and differentiation‐associated markers. HLA‐DR expression remained differentially regulated between subsets (*p* < 0.01), and significant differences were also observed for CCR5 (p < 0.001), CD86 (p < 0.05), and CD68 (*p* < 0.01). In contrast, TLR4 and CD69 exhibited either modest or nonsignificant differences after culture.

These findings indicate that, although in vitro culture induces partial phenotypic modulation, intrinsic divergence between Slan^+^ and Slan^−^ monocytes is not fully lost under unstimulated conditions. The persistence of subset‐specific expression patterns after 48 h supports the notion that Slan^+^ monocytes do not merely represent transient activation states of Slan^−^ cells but retain a distinct phenotypic identity in the absence of exogenous stimuli.

Taken together, the persistence of subset‐specific differences after 48 h under unstimulated conditions indicates that Slan^+^ and Slan^−^ monocytes maintain intrinsic phenotypic divergence beyond the immediate ex vivo state. However, the partial modulation observed for selected markers also suggests dynamic plasticity in vitro. Based on this balance between stability and adaptability, we next examined how each subset responds to defined inflammatory (LPS) and apoptotic (AB) stimuli. This approach allowed us to determine whether environmental cues amplify, attenuate, or reshape the baseline phenotypic distinctions between Slan^+^ and Slan^−^ monocytes.

**Figure 2:**
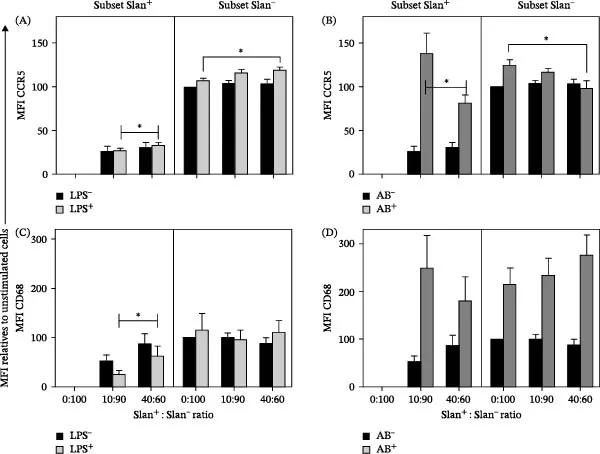
Surface expression markers related to monocyte differentiation under variable Slan^+^: Slan^−^ ratios and stimulation. CCR5 and CD68 expressions were assessed in reconstituted cultures stimulated with LPS or apoptotic bodies (ABs). (A, B) Median fluorescence intensity (MFI) of CCR5 with or without LPS (A), and with or without ABs (B). (C, D) CD68 MFI with or without LPS (C) and with or without ABs (D). Left panels: Slan^+^ monocytes; right panels: Slan^−^ monocytes. *n* = 5. Data are shown as mean ± SEM. Kruskal–Wallis test with Dunn’s multiple comparison post hoc test;  ^∗^ *p* < 0.05. Total number of independent experiments *n* = 5.

Surface expressions of CCR5 and CD68 were evaluated in response to ABs or LPS across varying Slan^+^:Slan^−^ ratios. LPS stimulation did not affect the surface expression of CCR5 in either Slan^+^ or Slan^−^ subsets. However, we observed a significant increase in CCR5 expression as the proportion of Slan^+^ cells increased in the coculture, affecting both Slan^+^ (*p* = 0.0375) and Slan^−^ (*p* = 0.0240) (Figure 2A), while AB stimulation caused a significant decrease in CCR5 expression. Notably, this effect was significantly more pronounced in cocultures containing a higher proportion of Slan^+^ monocytes (Slan^+^: *p* = 0.0379; Slan^−^: *p* = 0.0146; Figure 2B). CD68 expression increased significantly in Slan^+^ cells upon LPS exposure (*p* = 0.0236; Figure 2C), this increase was more pronounced as the proportion of Slan^+^ monocytes in the culture increased, whereas AB increased the surface expression of CD68 in both Slan^+^ and Slan^−^ monocytes; however, this effect was progressively reduced in Slan^+^ monocytes and enhanced in Slan^−^ monocytes as the proportion of Slan^+^ monocytes in the coculture increased (Figure 2D).

Importantly, the modulation of CCR5 and CD68 observed in these short‐term cultures reflects phenotypic changes associated with activation or polarization programs rather than full differentiation into macrophages or dendritic cells. Given the 48 h experimental timeframe and the absence of lineage‐defining transcriptional analyses, our data should be interpreted as surface marker modulation indicative of functional plasticity rather than terminal differentiation (Figure S4C).

#### 4.4.2. Activation Marker Profiles Under Inflammatory and Homeostatic Stimulus

**Figure 3:**
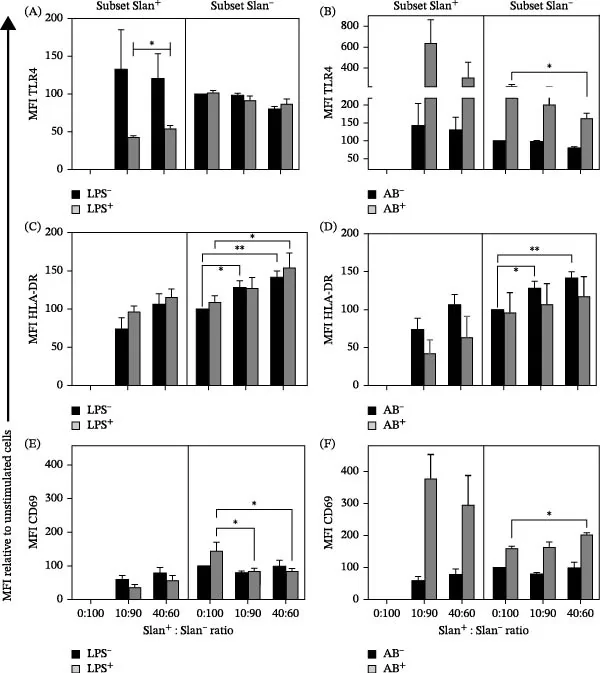
Activation marker expression in monocyte subsets under inflammatory or apoptotic stimulation. Surface expression of TLR4, HLA‐DR, and CD69 was assessed in Slan^+^ and Slan^−^ monocytes after 48 h of culture. (A, B) TLR4 MFI with or without LPS (A) and ABs (B). (C, D) HLA‐DR MFI with or without LPS (C) and ABs (D). (E, F) CD69 MFI with or without LPS (E) and ABs (F). Left panels: Slan^+^ monocytes; right panels: Slan^−^ monocytes. *n* = 5. Kruskal–Wallis test with Bonferroni‐adjusted Dunn’s post hoc test;  ^∗^ *p* < 0.05,  ^∗∗^ *p* < 0.005.

Upon LPS stimulation, Slan^+^ monocytes downregulated TLR4; however, this reduction was attenuated as the proportion of Slan^+^ monocytes increased in the coculture (*p* = 0.0106), while Slan^−^ monocytes exhibited a decreasing trend (Figure 3A). In contrast, AB increased TLR4 expression in both subsets, nevertheless, samples with a higher proportion of Slan^+^ monocytes showed decreased TLR4 expression in Slan^−^ monocytes (*p* = 0.0426; Figure 3B). LPS induced HLA‐DR upregulation in both subsets, an effect that was amplified in the presence of increased frequencies of Slan^+^ monocytes (Figure 3C). Conversely, AB treatment downregulated HLA‐DR expression, particularly in Slan^−^ monocytes, suggesting Slan^+^‐dependent modulation of antigen‐presenting capacity (Figure 3D). LPS and ABs treatment increased CD69 expression in Slan^−^ cells (*p* = 0.0108). However, increasing the proportion of Slan^+^ cells in the coculture had opposite effects depending on the stimulus: in LPS‐treated cultures, CD69 expression decreased as the proportion of Slan^+^ cells increased, whereas in ABs‐treated cultures, CD69 expression increased (*p* = 0.0062; Figure 3E,F).

Slan^+^ monocytes constitute a minor fraction of circulating monocytes in vivo (~5%–10% of total monocytes). Accordingly, a 9:1 (Slan^−^:Slan^+^) ratio was selected to approximate physiological predominance under steady‐state conditions. The 6:4 ratio modeled a relative enrichment scenario to evaluate whether shifts in subset balance modify functional outcomes. These ratios were chosen to explore changes in relative abundance rather than to reproduce a single fixed in vivo proportion.

#### 4.4.3. Costimulatory Molecule Modulation

**Figure 4:**
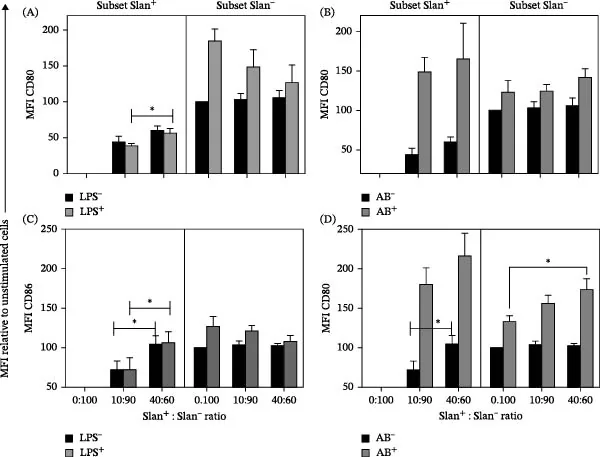
Modulation of costimulatory molecules CD80 and CD86 by Slan^+^:Slan^−^ ratio and stimulation. Expression of CD80 and CD86 in Slan^+^ and Slan^−^ monocytes after stimulation with LPS or ABs. (A, B) CD80 MFI with or without LPS (A) and with or without ABs (B). (C, D) CD86 MFI with or without LPS (C) and ABs (D). Left panels: Slan^+^ monocytes; right panels: Slan^−^ monocytes. *n* = 5. Kruskal–Wallis with Dunn’s test;  ^∗^ *p* < 0.05.

In the Slan^+^ subset, LPS induced a significant increase in CD80 expression at higher Slan^+^ proportions, particularly at the 40:60 ratio. (*p* = 0.0236; Figure 4A) In contrast, Slan^−^ cells showed higher basal CD80 expression, which was further enhanced by LPS. AB treatment markedly increased CD80 expression in both Slan^+^ and Slan^−^ subsets. This effect was more pronounced as the proportion of Slan^+^ monocytes increased, with significant differences observed at higher Slan^+^: Slan^−^ ratios (Figure 4B). CD86 expression also rose considerably in Slan^+^ cells with LPS, particularly at increased Slan^+^ proportions (*p* = 0.0379; Figure 4C) and AB treatment strongly enhanced CD86 expression in both subsets, with a progressive and significant increase in Slan^−^ cells as the proportion of Slan^+^ monocytes increased (*p* = 0.0353; Figure 4D). These findings suggest a subset‐ and stimulus‐dependent modulation of costimulatory pathways.

#### 4.4.4. Reciprocal Phenotypic Modulation Between Slan^+^ and Slan^−^ Monocytes in Reconstitution Cultures

To investigate whether relative subset composition influences monocyte phenotype, Slan^+^ and Slan^−^ monocytes were cocultured at graded proportions and analyzed after 48 h under unstimulated and stimulated conditions.

##### 4.4.4.1. Impact of Increasing Slan^+^ Proportion on Slan^−^ Monocytes

Under graded reconstitution conditions, increasing the proportion of Slan^+^ monocytes induced measurable modulation of activation‐associated markers within the Slan^−^ population. In particular, HLA‐DR, CD86, and CCR5 expression levels in Slan^−^ monocytes varied according to the relative abundance of Slan^+^ cells, suggesting subset‐dependent phenotypic adjustment.

These changes were further accentuated under LPS stimulation, where increasing Slan^+^ proportions amplified inflammatory‐associated marker expression in Slan^-^ monocytes.

In the presence of ABs, modulation patterns differed, indicating that environmental context shapes the direction and magnitude of inter‐subset influence.

##### 4.4.4.2. Impact of Increasing Slan^−^ Proportion on Slan^+^ Monocytes

Conversely, altering the relative abundance of Slan^−^ monocytes also resulted in phenotypic shifts within Slan^+^ cells. Changes in HLA‐DR, CD86, and CD69 expression across graded ratios suggest that regulatory interactions are not unidirectional.

Although the magnitude of modulation varied between markers and stimuli, the consistent ratio‐dependent trends observed across experiments support the concept of reciprocal phenotypic modulation between circulating monocyte subsets.

### 4.5. Cytokine Secretion Profiles

Cytokine accumulation in monocyte cultures varied with Slan^+^ cell proportion and stimulus. LPS significantly increased levels of TNF‐α, IL‐12, IL‐23, IL‐6, IL‐1β, and CCL2 (Figure 5A–G), with clear dose–response relationships. In AB‐stimulated cultures, similar trends were observed for TNF‐α, IL‐12, IL‐23, and CCL2, while IL‐6 and IL‐1β decreased slightly as Slan^+^ proportion increased. IL‐10 showed a nonlinear pattern, peaking at intermediate Slan^+^ proportions.

Figure 5Cytokine production in monocyte cultures under increasing Slan^+^:Slan^−^ ratios and stimulation. Monocyte subsets were combined at defined proportions (0:100, 10:90, 40:60 Slan^+^:Slan^−^; total 1 × 10^5^ cells/well) and stimulated with LPS (1 μg/mL), apoptotic bodies (five ABs/monocyte), or left unstimulated (NS) for 48 h. Supernatants were collected and analyzed using the Luminex 200 system. Concentrations of (A) TNF‐α, (B) IL‐23, (C) IL‐12, (D) IL‐6, (E) IL‐10, (F) IL‐1β, and (G) CCL2 were calculated from standard curves generated in each assay. Data represent five independent experiments performed with cells from five different donors (one donor per experiment). Each condition was measured in technical duplicate. Results are expressed as mean ± SEM. Statistical comparisons were performed using Kruskal–Wallis test followed by Dunn’s post hoc test with Bonferroni correction for multiple comparisons.  ^∗^ *p* < 0.05;  ^∗∗^ *p*  < 0.005;  ^∗∗∗^ *p* < 0.0005.

**Figure:**
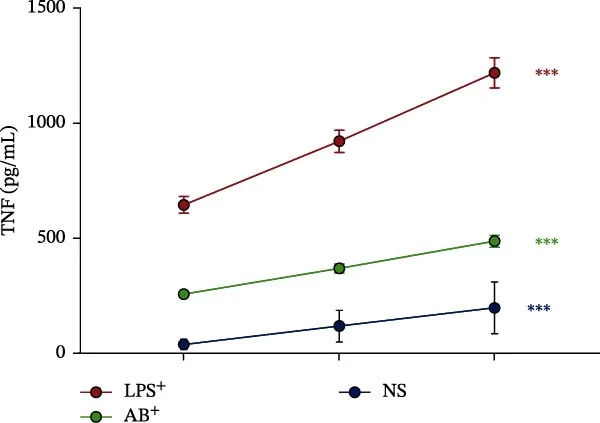
(A)

**Figure:**
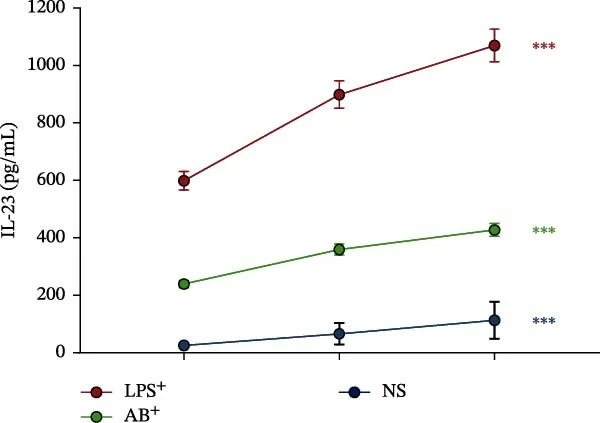
(B)

**Figure:**
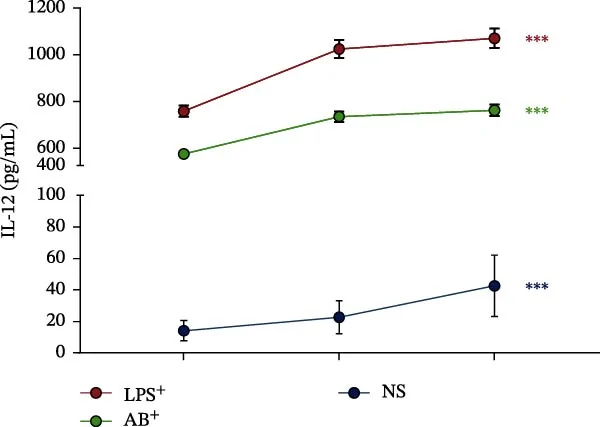
(C)

**Figure:**
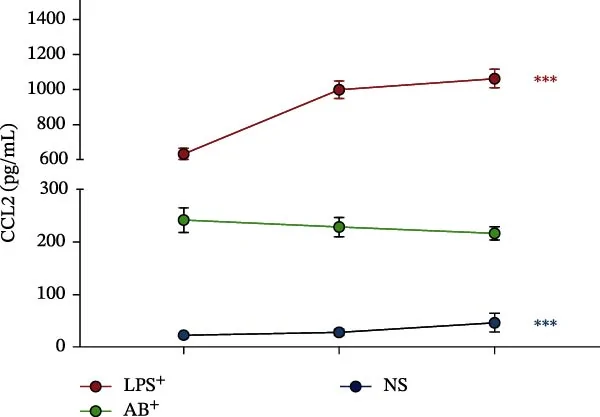
(D)

**Figure:**
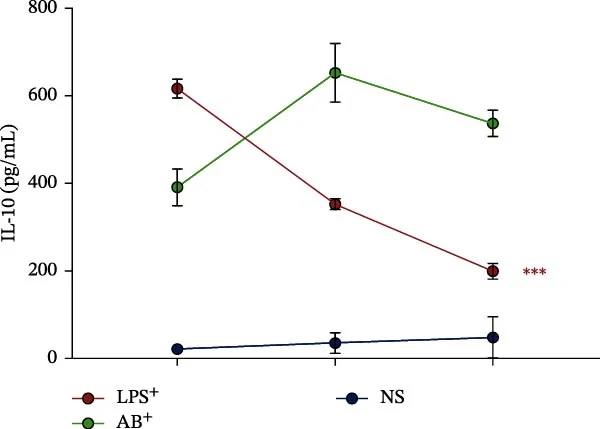
(E)

**Figure:**
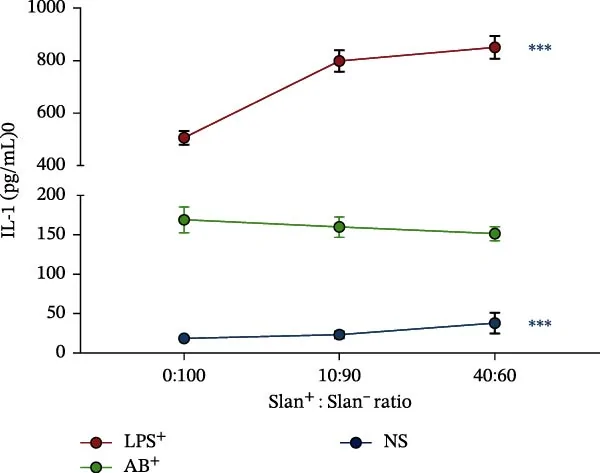
(F)

**Figure:**
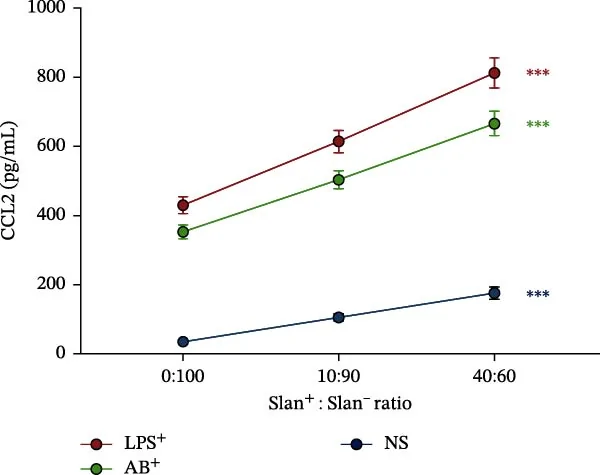
(G)

### 4.6. T Cell Proliferation Inhibition by Slan^+^ Monocytes

To directly address the comparative capacity of Slan^+^ and Slan^−^ monocyte subsets to modulate T cell responses, highly purified populations were sorted and reconstituted at defined ratios prior to coculture with autologous CD3^+^ T cells under identical experimental conditions. This experimental design allowed a direct functional comparison between subsets while preserving controlled total monocyte numbers, thereby isolating the specific contribution of Slan^+^ cells to T cell proliferation and cytokine modulation (Figure S4A).

In coculture experiments, increasing Slan^+^ proportions correlated with a reduction in CD4^+^ and CD8^+^ T cell proliferation, both in the absence and presence of AB (Figure 6). This inhibitory effect was more substantial in CD4^+^ cells and pronounced under ABs stimulation. PHA‐L‐stimulated T cells attenuated proliferation when cocultured with Slan^+^ monocytes, especially for CD4^+^ subsets (*p* = 0.0133 for 0: 100 vs., 40:60 ratios). The sorting strategy and basal activation status of Slan^+^ and Slan^−^ monocytes prior to coculture are shown in Figure S5, while the flow cytometry gating strategy used to quantify T cell proliferation is detailed in Figure S6.

Figure 6Proliferation of autologous CD3^+^ T cells in cocultures with monocyte subsets exposed to apoptotic bodies. Autologous CD3^+^ T lymphocytes were isolated by magnetic separation (>95% purity), labeled with CFSE, and cocultured with reconstituted monocyte mixtures at defined Slan^+^:Slan^−^ ratios (0:100, 10:90, 40:60; total monocytes 1 × 10^5^ cells/well) at a 3:1 lymphocyte‐to‐monocyte ratio. Cultures were maintained for 120 h at 37°C in 5% CO_2_. (A) Proliferation of CD4^+^ (left panels) and CD8^+^ (right panels) T cells in the absence of mitogenic stimulation (no PHA‐L), with or without apoptotic bodies (ABs; five ABs per monocyte). (B) Proliferation of CD4^+^ (left panels) and CD8^+^ (right panels) T cells in the presence of PHA‐L (5 μg/mL), with or without AB stimulation. After 120 h, cells were stained with Zombie Violet viability dye, anti‐CD4, and anti‐CD8 antibodies. Proliferation was assessed by CFSE dilution within viable CD4^+^ or CD8^+^ gated populations. Dividing cells were defined as those undergoing at least one cell division relative to undivided CFSE^high^ controls. Data are expressed as the percentage of divided cells. Results represent five independent experiments performed with cells from five different donors (one donor per experiment). Each condition was analyzed in technical duplicate. Data are shown as mean ± 95% CI. Statistical comparisons were performed using Kruskal–Wallis test followed by Dunn’s post hoc test with Bonferroni correction for multiple comparisons.  ^∗^Indicates *p* < 0.05.

**Figure:**
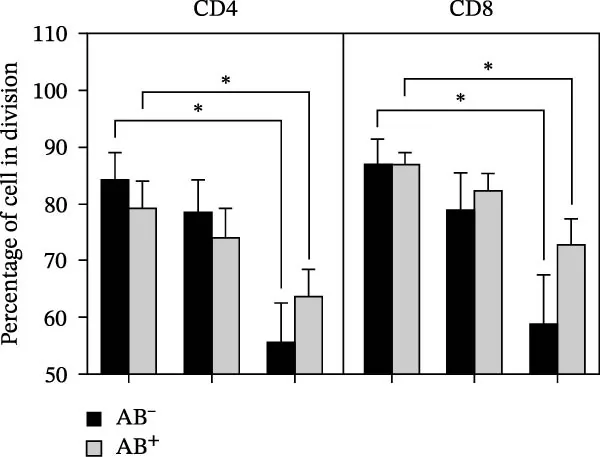
(A)

**Figure:**
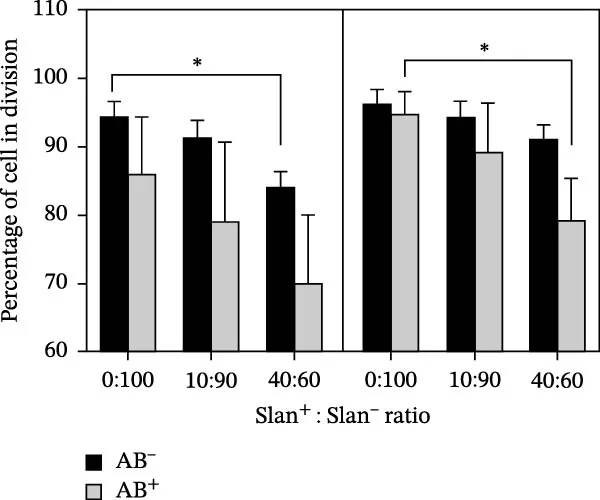
(B)

### 4.7. Cytokine Profiles in T Cell Cocultures

Supernatants from monocyte–T cell cocultures revealed decreased IL‐2, IL‐13, and IL‐10 production as Slan^+^ proportions increased, particularly in AB‐treated groups. In contrast, IL‐17 levels increased significantly with higher Slan^+^ ratios under PHA‐L and AB stimulation (*p* = 0.0006 for 0:100 vs. 40:60; Figure 7D). These data suggest that Slan^+^ monocytes may shape T cell responses by suppressing proliferation and modulating cytokine environments.

Figure 7Cytokine profile in cocultures of CD3^+^ T cells and monocytes under apoptotic stimulation. IL‐2 (A), IL‐13 (B), IL‐10 (C), and IL‐17 (D) levels measured in supernatants after 120 h of coculture at varying Slan^+^:Slan^−^ ratios. Cytokines assessed with or without ABs and PHA‐L. *n* = 5. Kruskal–Wallis with Dunn’s test;  ^∗^ *p* < 0.05,  ^∗∗^ *p* < 0.005,  ^∗∗∗^ *p* < 0.0005.

**Figure:**
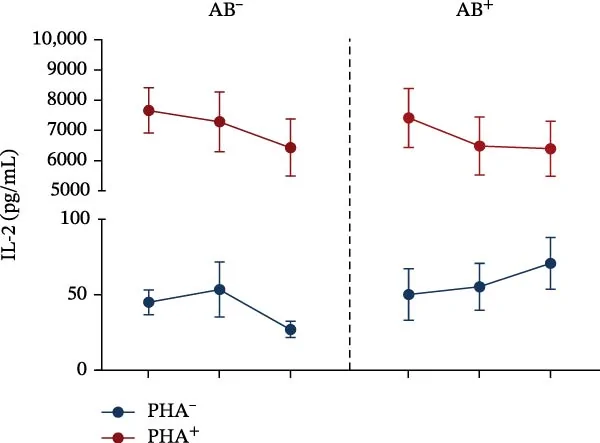
(A)

**Figure:**
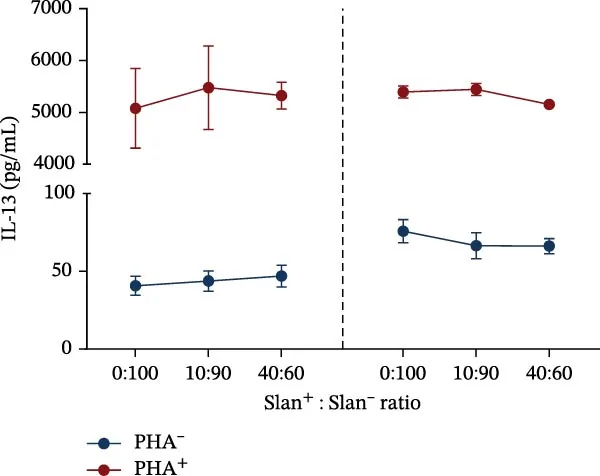
(B)

**Figure:**
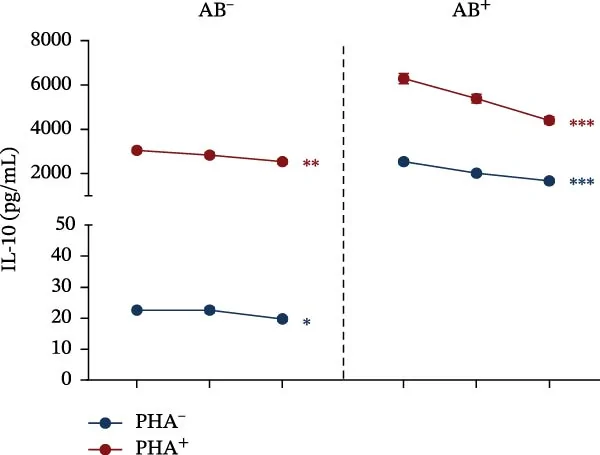
(C)

**Figure:**
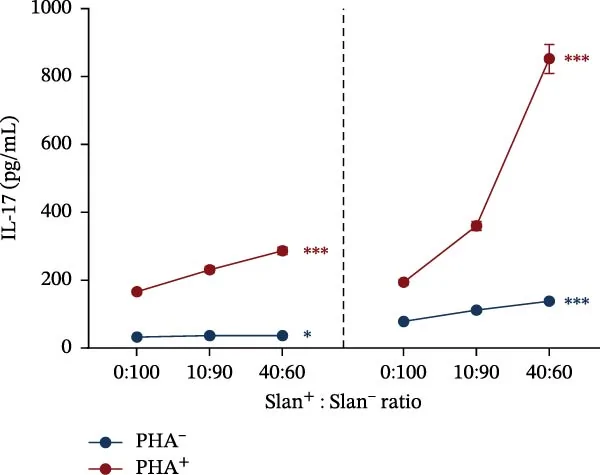
(D)

## 5. Discussion

Monocyte‐mediated regulation of immune responses is a well‐documented phenomenon across diverse physiological and pathological contexts [15–18]. Regulatory monocytes in healthy individuals can suppress T cell proliferation and cytokine production; however, this function is often impaired in chronic inflammatory diseases such as rheumatoid arthritis, where CD14^+^CD71^−^CD73^+^CD25^+^ monocytes show decreased IL‐10 production and insufficient immunoregulatory capacity [16].

Although some monocytes with regulatory functions have been categorized as myeloid‐derived suppressor cells (MDSCs), MDSCs are a heterogeneous population comprising immature precursors of granulocytes, macrophages, and dendritic cells [19]. In previous work, we showed that CD14^+^CD16^++^ monocytes can suppress T cell proliferation and modulate the response of other monocytes to apoptotic stimuli [11]. More recently, transcriptomic and phenotypic studies have confirmed that Slan^+^ cells are indeed nonclassical monocytes and not dendritic cells, as previously hypothesized [7, 20].

Here, we show that CD14^+^CD16^++^Slan^+^ monocytes influence classical CD14^++^CD16^−^ monocytes by modulating the expression of surface markers, cytokine profiles, and T cell responses. The presence of Slan^+^ monocytes increased HLA‐DR expression on Slan^−^ monocytes in a dose‐dependent manner, particularly in the presence of LPS [21]. This suggests that Slan^+^ cells may influence antigen presentation dynamics within the monocyte compartment.

TLR4 expression revealed differential regulation depending on both stimulus and subset. While Slan^+^ monocytes upregulated TLR4 in response to LPS, Slan^−^ cells exhibited decreased expression, a divergence possibly explained by distinct posttranscriptional regulatory mechanisms involving miR‐181a [22, 23]. Similarly, stimulation with ABs led to TLR4 downregulation and reduced IL‐6 and IL‐1β secretion in Slan^−^ monocytes, suggesting that Slan^+^ cells may confer anti‐inflammatory conditioning to neighboring subsets.

HLA‐DR expression increased significantly in the presence of Slan^+^ cells and LPS, particularly in Slan^−^ monocytes, which aligns with reports of enhanced HLA‐DR levels in CD16^+^ monocytes [7]. However, in clinical scenarios such as systemic inflammatory response syndrome (SIRS), HLA‐DR expression can be modulated by cortisol and IL‐10, with classical monocytes being more susceptible to downregulation [24]. Interestingly, TNF‐α has a dual role in HLA‐DR regulation, promoting or suppressing its expression depending on the cellular context and the involvement of CIITA or IFN‐γ pathways [25–27].

The observed upregulation of CCR5 and CD68 in Slan^+^ monocytes in response to LPS stimulation is consistent with macrophage polarization toward pro‐inflammatory M1 phenotypes. It suggests that Slan^+^ cells may acquire or induce differentiation markers under inflammatory conditions [28–30]. However, these markers decreased under AB stimulation, further supporting a regulatory or tolerogenic role for Slan^+^ monocytes in noninflammatory environments.

Costimulatory molecules CD80 and CD86 showed stimulus‐ and subset‐dependent patterns. Slan^+^ monocytes upregulated both molecules in response to LPS, while Slan^−^ cells showed reduced or unchanged expression. CD80 in particular is associated with CTLA‐4 binding and regulatory T cell (Treg) activity, which may suggest a dual role for Slan^+^ monocytes in both activation and immune resolution [31].

Cytokine analysis revealed strong interactions between Slan^+^ proportion and stimulus type. LPS promoted a robust M1/Th1 response with increased TNF‐α, IL‐12, IL‐23, and CCL2 secretion, while ABs also induced TNF‐α and IL‐12 but led to decreased IL‐1β and IL‐6 and a moderate rise in IL‐10. This pattern mirrors previous observations of dendritic cell maturation induced by ABs, which leads to transient suppression of inflammatory cytokines and upregulation of costimulatory molecules [32].

Interestingly, in T cell cocultures, higher proportions of Slan^+^ monocytes suppressed CD4^+^ and CD8^+^ proliferation and reduced IL‐2, IL‐13, and IL‐10 secretion, while IL‐17 levels rose. This suggests that Slan^+^ monocytes may favor Th17 differentiation over regulatory or Th2 responses in certain conditions [33–35]. Although ABs represent a homeostatic stimulus rather than an inflammatory trigger, the absence of inflammatory signals per se does not automatically confer regulatory capacity. In this study, the reduced T cell proliferation and the altered cytokine profile observed in the presence of Slan^+^ monocytes under homeostatic conditions suggest a diminished stimulatory function and a potential shift toward a regulatory phenotype. However, formal demonstration of regulatory activity would require dedicated suppression assays directly testing the ability of Slan^+^ monocytes to inhibit effector T cell responses in trans or in competitive settings. Therefore, our data support a context‐dependent modulatory role rather than a definitive regulatory function.

The immunomodulatory plasticity of Slan^+^ cells has been observed in tumor microenvironments, where they can acquire macrophage‐like or dendritic‐like phenotypes depending on the cytokine milieu. In multiple myeloma, Slan^+^ monocytes lose IL‐12 production and shift toward an intermediate phenotype when cocultured with tumor cells [36]. In renal cell carcinoma, however, these cells express IL‐10 and show tolerogenic behavior [37]. These findings underscore the microenvironmental sensitivity of Slan^+^ monocytes.

Although our experiments demonstrate robust effects after 48 h of culture, the temporal dimension of Slan^+^ cell plasticity remains underexplored. Longer stimulation may reveal different dynamics of marker expression or functional polarization, particularly in Slan^−^ monocytes that may require more time to respond [38]. The modest presence of CD14^-^CD16^+^Slan^−^ cells in the Slan^−^ fraction may have contributed to early activation in some conditions [39]. The present study also addressed the need for direct functional comparison between purified Slan^+^ and Slan^−^ subsets in T cell coculture systems. By employing sorted populations under identical experimental conditions, we demonstrate that Slan^+^ monocytes exert a distinct suppressive effect on CD4^+^ and CD8^+^ T cell proliferation. However, although we evaluated markers commonly associated with macrophage or dendritic cell programs, our experimental design does not support conclusions regarding complete lineage differentiation. Rather, the observed phenotypic changes likely represent context‐dependent activation states within the monocyte compartment.

While ABs used in our system were devoid of HMGB1 and free of endotoxins, the precise identity of the receptors mediating their recognition by Slan^+^ cells remain unclear. Scavenger receptors, including SREC‐I, may form complexes with TLR4 and influence cytokine responses [40, 41].

Finally, histamine and stress signals can also modulate Slan^+^ monocyte behavior via histamine receptors, particularly H4R, reducing pro‐inflammatory cytokine secretion and potentially affecting their capacity to stimulate T cells [42, 43]. These pathways merit further investigation in the context of inflammatory or autoimmune diseases.

## 6. Conclusions

This study demonstrates that CD14^+^CD16^++^Slan^+^ monocytes exert a distinct and dose‐dependent modulatory influence on classical CD14^++^CD16^−^ monocytes and autologous CD3^+^ T lymphocytes, particularly in the context of apoptotic and inflammatory stimuli. Slan^+^ monocytes displayed enhanced uptake of ABs, increased expression of activation and differentiation markers, and influenced cytokine production profiles in both monocyte subsets and cocultured lymphocytes.

Notably, Slan^+^ monocytes promoted the upregulation of HLA‐DR and CCR5 on classical monocytes, shaping cytokine landscapes toward either pro‐inflammatory (LPS) or regulatory (AB) profiles. They also suppressed the proliferation of CD4^+^ and CD8^+^ T cells in vitro and modulated T cell cytokine signatures, including a shift toward IL‐17 production. These findings suggest a dual functional identity for Slan^+^ monocytes, which can promote either inflammatory or tolerogenic responses, depending on the context and cellular interactions.

Importantly, these results provide mechanistic insights into the potential contribution of Slan^+^ monocytes to immunopathology in systemic autoimmune diseases. In lupus nephritis, for instance, increased infiltration of CD16^+^Slan^+^ monocytes has been observed in glomerular tissue, where they may contribute to immune complex‐driven inflammation and tissue damage. Our findings of enhanced pro‐inflammatory cytokine production and monocyte activation in the presence of Slan^+^ cells, especially under LPS stimulation, suggest that these monocytes could play a pathogenic role in the inflammatory milieu of lupus nephritis, while retaining the potential to exert regulatory effects in tolerogenic settings.

Taken together, our data support a context‐dependent regulatory capacity of Slan^+^ monocytes rather than a fixed inflammatory phenotype, highlighting their plasticity within the human monocyte compartment.

### 6.1. Data Limitations and Perspectives

This study was performed using an in vitro reconstitution model with purified monocyte subsets obtained from healthy donors. Although this approach allowed controlled manipulation of Slan^+^:Slan^−^ ratios and direct assessment of subset‐specific interactions, it does not fully recapitulate the complexity of tissue microenvironments where additional stromal, endothelial, and lymphoid signals contribute to monocyte plasticity.

Our experiments were conducted at defined time points (48 h for monocyte assays and 120 h for T cell cocultures), which may not capture the full temporal dynamics of Slan^+^ monocyte polarization. Longer exposure or sequential stimulation models could reveal additional regulatory or inflammatory transitions.

Mechanistically, although changes in surface markers and cytokine profiles were clearly observed, intracellular signaling pathways and transcriptional programs were not directly evaluated. Therefore, the molecular mechanisms underlying the modulatory effects of Slan^+^ monocytes remain to be elucidated.

Furthermore, T cell functional skewing was inferred from proliferation and cytokine secretion, but intracellular cytokine staining and lineage‐defining transcription factors were not assessed.

Future studies integrating transcriptomic profiling, metabolic analysis, and in vivo validation in disease contexts such as SLE will be essential to clarify whether Slan^+^ monocytes act predominantly as inflammatory amplifiers or regulatory modulators depending on environmental cues.

### 6.2. Study Considerations Regarding the AB Source

We acknowledge that ABs derived from transformed cell lines could, in principle, carry immunostimulatory signals. To mitigate this, we used K562 cells because they display negligible basal HLA class I/II expression, thereby limiting alloantigen‐related confounding in our autologous monocyte–T cell cocultures. Moreover, our AB preparation was enriched from DiOC6^low^ PI^−^ events and was HMGB1‐negative after thawing, supporting an apoptotic (rather than necrotic) profile. Future studies may compare K562‐derived ABs with donor‐derived apoptotic material; however, in our hands, autologous neutrophil‐ or T cell‐derived preparations introduced substantial heterogeneity in death modalities and required larger blood volumes and/or multiple collections, limiting feasibility and reproducibility for a donor‐matched design.

## Author Contributions

Cristian A. Anacona and Karen Álvarez conducted experiments and analyzed data. Cristian A. Anacona performed cell sorting experiments and cocultures. Gloria Vásquez contributed to the study design and interpretation. Mauricio Rojas conceived the study, was the PI of the project, supervised the project, and wrote the manuscript.

## Funding

Financial support from MINCIENCIAS through the Contingent Recovery Financing Contract Number 919 of 2019, signed on December 19, 2019, for the project: “*Expresión diferencial de genes en Monocitos SLAN+/− tratados o no con micropartículas de pacientes con Lupus eritematoso sistémico con y* sin *nefritis. Una evidencia de la capacidad moduladora de estas vesículas extracelulares,”* Code 111584467246, Contract Number RC Number 919 de 2019. Additional funding support was provided through the Immunology and Immunogenetics Research Group (GICIG).

## Disclosure

All authors reviewed and approved the final version.

## Conflicts of Interest

The authors declare no conflicts of interest.

## Supporting Information

Additional supporting information can be found online in the Supporting Information section.

## Supporting information


**Supporting Information** Figure S1. Flow cytometry gating strategy used for the identification of human monocyte subsets and Slan⁺ monocytes in PBMCs. Supporting Figure S2. Basal ex vivo phenotypic characterization of circulating monocyte subsets in whole blood, including HLA‐DR, TLR4, CCR5, CD86, CD69, and CD68 expression. Supporting Figure S3. Phenotypic profile of Slan^+^ and Slan^−^ monocytes after 48 h under unstimulated culture conditions. Supporting Figure S4. Functional comparison of Slan^+^ and Slan^−^ monocytes in T cell coculture, including T cell proliferation analysis and assessment of monocyte‐derived dendritic cell markers. Supporting Figure S5. Sorting strategy, postsort purity and recovery, and evaluation of the basal activation status of Slan^+^ and Slan^−^ monocytes before and after FACS isolation. Supporting Figure S6. Flow cytometry gating strategy used to analyze proliferation of sorted T lymphocytes cocultured with Slan^+^ monocytes. Si quiere una versión un poco más formal, más parecida al estilo Wiley, también puede usar esta: Supporting Information is available online and includes six supporting figures. Supporting Figure S1 shows the gating strategy for identification of human monocyte subsets and Slan^+^ monocytes. Supporting Figure S2 presents the basal ex vivo phenotypic characterization of circulating monocyte subsets in whole blood. Supporting Figure S3 shows the phenotypic profile of Slan^+^ and Slan^−^ monocytes after 48 h under unstimulated culture conditions. Supporting Figure S4 provides the functional comparison of Slan^+^ and Slan^−^ monocytes in T cell coculture and the assessment of monocyte‐derived dendritic cell markers. Supporting Figure S5 shows the sorting strategy, postsort purity and recovery, and basal activation status of Slan^+^ and Slan^−^ monocytes before and after FACS isolation. Supporting Figure S6 presents the gating strategy for the analysis of proliferation of sorted T lymphocytes cocultured with Slan^+^ monocytes.

## Data Availability

The data that support the findings of this study are available upon request from the corresponding author. The data are not publicly available due to privacy or ethical restrictions.
