# Supplementary material for: Regulatory Effects of CD14+CD16++Slan+ Monocytes on Classical Monocytes and T Lymphocytes in Response to Inflammatory and Phagocytic Stimuli
Source: J Immunol Res. 2026 Apr 11;2026:3322498. doi: 10.1155/jimr/3322498 (PMC13581061; doi:10.1155/jimr/3322498)
Supplement: Supplementary file 1 [file JIMR-2026-3322498-s001.docx]

**Supplementary Figure S1**. **Flow cytometry gating strategy for the identification of human monocyte subsets and Slan⁺ monocytes.** Peripheral blood mononuclear cells (PBMCs) were analyzed by multiparametric flow cytometry to identify monocyte subsets and the Slan⁺ population. (**A**) Viable cells were first selected by excluding dead cells stained with the viability dye SYTOX Red using SSC-A versus SYTOX Red. (**B**) PBMCs were subsequently identified according to their forward and side scatter characteristics (FSC-A vs SSC-A). (**C**) Doublets were excluded by gating singlet cells using FSC-A versus FSC-H parameters. (**D**) Within the singlet population, HLA-DR⁺ cells displaying monocyte-like scatter properties were selected to exclude lymphocytes and other non-monocytic populations. (**E**) Monocytes were further identified based on CD14 and CD16 expression. (**F**) Monocyte subsets were defined according to the current consensus classification as classical monocytes (CD14⁺⁺CD16⁻), intermediate monocytes (CD14⁺⁺CD16⁺), and non-classical monocytes (CD14⁺CD16⁺⁺). (**G**) Slan⁺ monocytes were identified within the CD16⁺ monocyte compartment based on the expression of the 6-sulfo LacNAc (Slan) epitope. Percentages shown in each panel indicate the frequency of cells within the parent population.

**Supplementary Figure S2. Basal ex vivo phenotypic characterization of circulating monocyte subsets in whole blood.**

**(A)** Representative histograms showing the basal surface expression of HLA-DR, TLR4, CCR5, CD86, CD69, and CD68 in classical (CD14⁺⁺CD16⁻Slan⁻; beige), CD16⁺Slan⁻ (red), and CD14⁺CD16⁺⁺Slan⁺ (blue) monocytes from healthy donors. Histograms were generated from gated monocyte populations identified by forward and side scatter properties followed by CD14/CD16/Slan expression. Fluorescence intensity is displayed on a logarithmic scale.

**(B)** Quantitative analysis of marker expression shown as median fluorescence intensity (MFI ± 95% confidence interval) for each subset. Statistical comparisons were performed using Kruskal–Wallis test followed by Dunn’s multiple comparisons test with Bonferroni correction. Significance levels are indicated as *p* < 0.05 (*) and *p* < 0.01 (**).

Whole blood samples were obtained from four healthy donors and analyzed directly ex vivo to assess steady-state phenotypic differences among circulating monocyte subsets.

**Supplementary Figure S3. Phenotypic profile of Slan⁺ and Slan⁻ monocytes after 48 h under unstimulated culture conditions.**

Slan⁺ and Slan⁻ monocytes were cultured for 48 h in the absence of exogenous stimulation (NS condition), and the expression of activation- and differentiation-associated surface markers was analyzed by flow cytometry. Bar graphs represent median fluorescence intensity (MFI) ± 95% confidence interval for HLA-DR, TLR4, CCR5, CD86, CD69, and CD68.

After 48 h in culture, significant differences between Slan⁺ and Slan⁻ monocytes were observed for HLA-DR (**p < 0.01**), CCR5 (***p < 0.001***), CD68 (**p < 0.01**), and TLR4 (*p < 0.05*), whereas CD86 and CD69 did not show statistically significant differences between subsets. Statistical comparisons were performed using the Kruskal–Wallis test followed by Dunn’s post hoc test with Bonferroni correction. Significance levels are indicated as *p* < 0.05 (*), p < 0.01 (****), and p < 0.001 (***).

Data represent independent experiments performed with cells from healthy donors (n = 4–5).

**Supplementary Figure S4. Functional comparison of Slan⁺ and Slan⁻ monocytes in T-cell co-culture and assessment of monocyte-derived dendritic cell markers.**

**(A)** Representative CFSE dilution histograms of CD4⁺ (blue) and CD8⁺ (red) T cells cultured for 96 h in the absence of stimulation (NS), or in co-culture with purified Slan⁻ or Slan⁺ monocytes in the presence of PHA. T cells were sorted by FACS (purity >98%), labeled with CFSE, and co-cultured with FACS-purified Slan⁺ or Slan⁻ monocytes (purity >95%). After 96 h, cells were stained with anti-CD8α, and dead cells were excluded using 7-AAD. Proliferation was quantified based on CFSE dilution within viable CD4⁺ and CD8⁺ T-cell gates.

**(B)** Consolidated analysis of four independent experiments showing the percentage of divided CD4⁺ and CD8⁺ T cells under each condition: total monocytes, Slan⁺ monocytes, and Slan⁻ monocytes. Data are presented as mean ± 95% confidence interval. Percentages represent cells undergoing at least one division as determined by CFSE dilution.

**(C)** Assessment of monocyte-derived dendritic cell (moDC) markers after 96 h of co-culture. A fraction of the cultured cells was stained for CD33 to identify myeloid cells and subsequently analyzed for expression of CD209 (DC-SIGN) and CD1a, canonical markers associated with moDC differentiation. Representative histograms are shown for total monocytes (orange), Slan⁻ monocytes (red), Slan⁺ monocytes (blue), and fluorescence-negative controls (green). No upregulation of CD209 or CD1a was detected in any monocyte subset, indicating absence of detectable moDC differentiation under the culture conditions used.

**Supplementary Figure S5. Sorting strategy and assessment of basal activation status of Slan⁺ and Slan⁻ monocytes prior to FACS isolation.**

**(A)** Representative gating strategy used to identify and sort Slan⁺ and Slan⁻ monocyte subsets from PBMC. Mononuclear cells were first gated based on FSC/SSC characteristics, followed by identification of CD14/CD16-defined monocyte populations. Slan expression (6-sulfo LacNAc) was then used to discriminate Slan⁺ from Slan⁻ fractions within the CD16 compartment prior to sorting on a FACS Aria III. Representative contour plots illustrate the sequential gating strategy.

**(B–D)** Basal expression levels of activation markers were evaluated in pre-sort gated populations to assess potential activation induced by antibody labeling and sorting procedures. Mean fluorescence intensity (MFI ± 95% CI) of **CD69 (B)**, **HLA-DR (C)**, and **CD86 (D)** were measured before and immediately after cell sorting. No significant differences were observed between PRE and POST conditions in either Slan⁺ or Slan⁻ subsets across independent experiments, indicating that the sorting procedure did not induce detectable activation.

Data represent independent biological replicates (n = 3–4 experiments performed explicitly to evaluate sorting-associated activation effects).

Post-sort purity exceeded **94% for Slan⁻ monocytes** and **89% for Slan⁺ monocytes**, with recovery efficiencies above **90% in both populations**.

Data represents independent donors (n = 4). Paired comparisons were performed using a two-tailed Wilcoxon matched-pairs signed-rank test; no significant differences were observed. No significant differences were observed between pre- and post-sort conditions for HLA-DR (p = 0.56), CD69 (p = 0.71), or CD86 (p = 0.48). Importantly, variability across donors was low, and no consistent directional trend toward activation was observed following sorting.

**Supplementary Figure S6. Flow cytometry gating strategy for the analysis of proliferation of sorted T lymphocytes co-cultured with Slan⁺ monocytes.** Previously purified T lymphocytes were labeled with CFSE and co-cultured with Slan⁺ monocytes. Proliferation of T-cell subsets was evaluated by flow cytometry using the following gating strategy. (A) Lymphocytes were first identified according to their forward and side scatter characteristics (FSC-A vs SSC-A), allowing the exclusion of debris and residual monocytes. (B) Doublets were excluded by selecting singlet cells based on FSC-A versus FSC-H parameters. (C) Viable cells were selected by excluding 7-AAD–positive events. (D) T-cell subsets were identified based on CD8 expression, allowing discrimination of CD8⁺ T cells and CD8⁻ cells (corresponding predominantly to CD4⁺ T cells). (E) T-cell proliferation was assessed by CFSE dilution within each subset, with representative histograms showing CFSE intensity in CD4⁺ (blue) and CD8⁺ (red) T cells.
